# Managing Febrile Respiratory Illnesses during a Hypothetical SARS Outbreak

**DOI:** 10.3201/eid1102.040524

**Published:** 2005-02

**Authors:** Kamran Khan, Peter Muennig, Michael Gardam, Joshua Graff Zivin

**Affiliations:** * St. Michael's Hospital, Toronto, Ontario, Canada;; † Columbia University, New York, New York, USA;; ‡ University Health Network, Toronto, Ontario, Canada

**Keywords:** Severe Acute Respiratory Syndrome, Influenza-Like-Illness, Influenza Vaccination, Mass Screening, Cost-Benefit Analysis, Human, perspective

## Abstract

Since the World Health Organization declared the global outbreak of severe acute respiratory syndrome (SARS) contained in July 2003, new cases have periodically reemerged in Asia. This situation has placed hospitals and health officials worldwide on heightened alert. In a future outbreak, rapidly and accurately distinguishing SARS from other common febrile respiratory illnesses (FRIs) could be difficult. We constructed a decision-analysis model to identify the most efficient strategies for managing undifferentiated FRIs within a hypothetical SARS outbreak in New York City during the season of respiratory infections. If establishing reliable epidemiologic links were not possible, societal costs would exceed $2.0 billion per month. SARS testing with existing polymerase chain reaction assays would have harmful public health and economic consequences if SARS made up <0.1% of circulating FRIs. Increasing influenza vaccination rates among the general population before the onset of respiratory season would save both money and lives.

## Abstract 2

Optimal management of febrile respiratory illnesses during a hypothetical SARS outbreak varies depending on a number of conditions, but increasing influenza vaccination rates would save money and lives.

On July 5, 2003, the World Health Organization (WHO) declared that human chains of transmission of severe acute respiratory syndrome (SARS) had ended. Since then, new cases of SARS have resurfaced in Asia, including several in the absence of laboratory exposures. This reemergence of the SARS-associated coronavirus (SARS-CoV) has sparked international concern and has prompted heightened surveillance by hospitals and health officials worldwide. Such concerns have been amplified by fears that a future SARS outbreak could coincide with respiratory infection season, when influenza infections and other febrile respiratory illnesses (FRIs) develop in large segments of the population.

Current SARS case-definition and case-exclusion criteria encompass clinical, epidemiologic, and laboratory features (*1*). Should the timely establishment of epidemiologic links between SARS cases be lost in a future outbreak, frontline healthcare providers would be forced to rely on clinical signs and symptoms or diagnostic testing to confirm or exclude infections with SARS-CoV (*2*). Unfortunately, the signs and symptoms of SARS are nonspecific and cannot be used reliably to differentiate SARS from other FRIs. Moreover, existing serologic tests for SARS-CoV cannot definitively exclude infection until at least 4 weeks has elapsed from the onset of symptoms and thus have no role in early clinical decision making (*1*). Although reverse transcriptase–polymerase chain reaction (RT-PCR) assays used to detect SARS-CoV can provide test results within a matter of hours, their suboptimal sensitivity makes them inadequate for ruling out SARS (*3*). Furthermore, since SARS infections would likely make up a minute fraction of FRIs circulating among the general population, the pretest probability, and thus the positive predictive value of RT-PCR tests, would be extremely low, even if future generation assays had better test sensitivity and specificity.

In 2003 and 2004, the emergence of SARS-CoV in China coincided with respiratory illness season, which suggests that the virus may resurface during winter months, like many other respiratory pathogens. Should this seasonal pattern recur, rapidly and accurately differentiating SARS infections from other FRIs would become a critical component of any future outbreak containment efforts (*2**,**3*). This distinction will also continue to be an important issue among travelers in whom FRIs develop after their return from SARS-affected areas. However, existing diagnostic limitations place frontline healthcare practitioners in a precarious position, since clinical decisions with potentially dangerous consequences must be made in the face of uncertainty. Recognizing such limitations, WHO recently called for the development of evidence-based clinical algorithms to help address these diagnostic dilemmas (*4*).

## Methods

### Overview and Definitions

A hypothetical cohort comprising all residents of New York City was entered into a decision-analysis model. The model is premised on a SARS outbreak during respiratory season where person-to-person transmission of SARS is documented and epidemiologic links between cases are poorly defined. The outbreak was designed to be consistent in size and duration with the Toronto outbreak (*5*). The analytic horizon of the analysis was defined as the expected lifetime of persons living in New York City during the 2004–2005 respiratory illness season. FRIs are defined herein as nonspecific infections caused by pathogens other than SARS-CoV for which the microbiologic origin cannot be determined on the basis of clinical grounds alone. The model was designed to identify the most effective and cost-effective uses of societal resources in managing FRIs of undetermined origin during a SARS outbreak.

The analysis was conducted in adherence with the reference case scenario as defined by the Panel on Cost-Effectiveness in Health and Medicine (*6*). All relevant costs and benefits were considered from the societal perspective of New York City, including those related to secondary transmission of SARS. Since costs and changes in health-related quality of life in the analysis were limited to a single respiratory season, no discounting was performed on these 2 parameters. However, all future years of life lost due to premature death from infections were discounted at an annual rate of 3%.

### Decision-Analysis Model

A decision-analysis model was constructed by using DATA 4.0 (TreeAge Software, Williamstown, MA, USA) that examined 2 competing strategies in the context of a SARS outbreak coinciding with respiratory season: 1) home isolation for persons with FRIs of undetermined origin, pending fever and symptom resolution for at least 24 hours and 2) outpatient diagnostic testing of FRIs to ascertain a microbiologic diagnosis with subsequent test-driven management. A third complementary strategy entailing mass influenza vaccination among the general population before the onset of respiratory season was considered in conjunction with the above competing strategies.

Primary assumptions of the model were as follows: 1) epidemiologic linkages between SARS cases are not well defined; 2) SARS cannot reliably be distinguished from other FRIs on clinical grounds alone; 3) current SARS tests cannot definitively rule out infection early in the course of illness (*1**,**7*); 4) public nonadherence to home isolation guidelines during a SARS outbreak would be negligible (*5**,**8*); 5) positive SARS (RT-PCR) test requires isolation precautions pending confirmation of the diagnosis (*2*); 6) patients with confirmed SARS cases will be managed as inpatients pending resolution of the clinical illness; 7) patients with confirmed SARS cases require isolation precautions for 10 days after resolution of illness (*2*); 8) persons with FRIs of undetermined origin must be afebrile and symptom-free for 24 hours before returning to work; 9) negative SARS (RT-PCR) test alone will have no influence on SARS isolation precautions (*2*); 10) negative SARS (RT-PCR) test result combined with a positive test for another respiratory pathogen will result in the discontinuation of SARS isolation precautions (*2*); 11) in the absence of appropriate isolation precautions, persons with SARS will transmit infection to 3 additional persons (*9**,**10*); 12) SARS, influenza, respiratory syncytial virus, and community-acquired pneumonia are the primary causes of death from FRIs; 13) a future SARS outbreak would be managed by using existing healthcare infrastructure; and 14) no proven effective treatment for SARS currently exists.

A plausible range of high and low values for each variable was used to conduct sensitivity analyses, which examined the influence of parameter error on the results of the analysis. Selected variables in the model are listed in Tables 1 and 2.

**Table 1:** Selected costs in the decision-analysis model*

Costs†	Low	Base	High	Source
Vaccines and medications				
Influenza vaccine	$10.00	$27.78	$40.00	11
Antibiotics for FRI‡	$30.00	$64.72	$80.00	12
Medical care§				
Ambulatory clinic visit	$40.00	$60.03	$80.00	13
Hospitalization for FRI	$5,000	$11,645	$15,000	14
Hospitalization for influenza	$7,500	$17,465	$25,000	14
Hospitalization for PUI	$15,000	$19,441	$25,000	14
Hospitalization for SARS	$20,000	$28,502	$40,000	14,15
Diagnostic tests				
Rapid influenza test	$15.00	$26.86	$40.00	16
Multiplex¶ RT-PCR	$50.00	$154.02	$200.00	Prodesse Inc., pers. comm.
SARS# RT-PCR	$20.00	$54.80	$100.00	Prodesse Inc., pers. comm.
Miscellaneous				
Patient time (per hour)	$15.00	$24.55	$30.00	17
Contact investigation (per SARS contact)	$100.00	$222.94	$300.00	5,18

*FRI, febrile respiratory illness; PUI, person under investigation (for SARS); SARS, severe acute respiratory syndrome; RT-PCR, reverse transcription–polymerase chain reaction. †Medical and nonmedical costs were adjusted to 2004 U.S. dollars by using the Consumer Price Index."? ‡Antimicrobial drug costs are based on a 7-day course of oral levofloxacin. §Includes laboratory tests, transportation costs, and patient time. ¶Detects influenza viruses A and B, respiratory syncytial viruses A and B, parainfluenza viruses 1–3, human metapneumovirus, *Legionella pneumophila*, *L. micdadei*, *Mycoplasma pneumoniae*, *Chlamydia pneumoniae*, and *Bordetella pertussis*. #Detects SARS-associated coronavirus and coronaviruses OC43 and 229E.

**Table 2:** Selected probabilities in the decision-analysis model*

Selected probabilities	Low	Base	High	Source
Diagnostic tests				
Sensitivity of influenza test	0.50	0.70	0.90	19
Specificity of influenza test	0.80	0.95	0.99	19
Sensitivity of multiplex† RT-PCR	0.70	0.85	0.95	20–22
Specificity of multiplex† RT-PCR	0.80	0.987	0.99	20–22
Sensitivity of SARS‡ RT-PCR	0.25	0.70	0.95	4,23
Specificity of SARS‡ RT-PCR	0.95	0.994	1.00	23,24
Morbidity and mortality				
Hospitalization due to influenza	0.002	0.004	0.01	25
Death due to influenza	0.0	0.0012	0.01	25,26
Hospitalization due to FRI	0.010	0.015	0.02	Calculated
Death due to FRI	0.0	0.0009	0.01	Calculated
Death due to SARS	0.10	0.15	0.20	24
Miscellaneous probabilities				
Probability of an FRI	0.10	0.33	0.50	27
Due to influenza	0.20	0.33	0.50	11,25,27,28
Due to multiplex† organisms other than influenza	0.20	0.33	0.50	29,30
Due to other causes§	0.20	0.33	0.50	Calculated
Due to SARS	0.0	0.0001	0.01	Assigned
Influenza vaccine effectiveness	0.35	0.67	0.85	31
Annual probability of poor match between vaccine and circulating influenza strains	0.05	0.20	0.50	31
Probability of successful self-care management of an FRI at home	0.33	0.67	1.00	Assumption
Probability of receiving outpatient antimicrobial drugs for an FRI	0.33	0.67	1.00	32
Miscellaneous values				
Patient time for outpatient medical visit (min)	30	50	90	Estimate
Influenza length of illness (d)	3	7	10	31
Other FRI¶ length of illness (d)	1	3	5	Estimate
Average duration of hospitalization, influenza (d)	5	10.2	15	14
Average duration of hospitalization, FRI¶ (d)	3	7.7	10	14
Average duration of hospitalization, SARS (d)	10	16	30	15
HRQL scores				
SARS, hospitalized	0.05	0.160	0.50	HUI
SARS, outpatient	0.25	0.670	0.75	HUI
SARS, contact	0.50	0.785	0.95	HUI
FRI, hospitalized	0.25	0.511	0.75	HUI
FRI, outpatient	0.50	0.804	0.95	HUI
Reproductive number for SARS#	2	3	4	9,10
Contact investigations (per SARS case)	25	50	100	5

*RT-PCR, reverse transcriptase–polymerase chain reaction; SARS, severe acute respiratory syndrome; FRI, febrile respiratory illness; HRQL, health-related quality of life; HUI, Health Utilities Index. †Refers to influenza viruses A and B, respiratory syncytial viruses A and B, parainfluenza viruses 1–3, human metapneumovirus, *Legionella pneumophila*, *L. micdadei*, *Mycoplasma pneumoniae*, *Chlamydia pneumoniae*, and *Bordetella pertussis*. ‡Detects SARS-associated coronavirus and coronaviruses OC43 and 229E. §Febrile respiratory illnesses not due to SARS, influenza viruses A and B, respiratory syncytial viruses A and B, parainfluenza viruses 1–3, human metapneumovirus, *L. pneumophila*, *L. micdadei*, *M. pneumoniae*, *C. pneumoniae*, and *B. pertussis*. ¶Febrile respiratory illnesses not due to SARS or influenza viruses A and B. #In the absence of public health interventions.

### Composition of FRIs

We used nationally representative data (*25**,**27*) in conjunction with studies published in the medical literature (*11**,**28**–**30*) to derive our base estimates for an "average" respiratory season. In our model, the microbiologic origin of an FRI was categorized into 1 of 4 mutually exclusive groups: 1) SARS-CoV and coronaviruses OC43 and 229E; 2) influenza viruses A and B; 3) a panel of common respiratory pathogens, including respiratory syncytial viruses A and B, parainfluenza viruses 1–3, human metapneumovirus, *Bordetella pertussis*, *Chlamydia pneumoniae*, *Mycoplasma pneumoniae*, *Legionella pneumophila*, and *L. micdadei*; and 4) all other causes.

In our base-case analysis, we assigned the proportion of FRIs due to SARS to be 0.01%, which was estimated assuming a SARS outbreak of similar size and duration to the Toronto outbreak. The proportion of FRIs due to influenza was derived from 2 large observational studies conducted over multiple respiratory seasons (*11**, **28*) and was corroborated by dividing the expected proportion of the U.S. population who get influenza each season (*25*) by the proportion of the U.S. population having influenzalike infections (*27*). The proportion of FRIs due to the common respiratory pathogen panel listed above was estimated from the medical literature (*29**,**30*). In our base-case scenario, we estimated that approximately one third of FRIs would be due to influenza, one third would be due to the panel of common respiratory pathogens, and the remaining one third would be due to other miscellaneous pathogens not indicated above.

### Diagnostic Tests

We evaluated 3 categories of rapid diagnostic tests with optimal turnaround times of <24 hours. The first category constitutes RT-PCR assays capable of detecting SARS-CoV as well as coronaviruses OC43 and 229E (*23**,**24*). A second category includes 2 multiplex PCR assays, which, when used in combination, can detect 13 different respiratory pathogens, including influenza viruses A and B, respiratory syncytial viruses A and B, parainfluenza viruses 1–3, human metapneumovirus, *C. pneumoniae*, *M. pneumoniae*, *L. pneumophila*, *L. micdadei*, and *B. pertussis* (*20**–**22*). The third category comprises a widely available enzyme immunoassay capable of rapidly detecting infections with influenza A and B (*19*).

The sensitivity and specificity of these tests were obtained from the medical literature (*19**–**24*), while the positive predictive value of each diagnostic test was calculated by incorporating the estimated prevalence of specific pathogens into Bayes' equation.

### Influenza Vaccination

The effectiveness of the influenza vaccine was derived from the medical literature (*31*). To account for seasonal variation between circulating strains of influenza and the composition of the trivalent vaccine, we varied the effectiveness of the vaccine over a wide range of plausible values in our sensitivity analysis. The average seasonal effectiveness of the influenza vaccine was adjusted by assuming that the vaccine would be poorly matched to circulating influenza strains approximately twice every 10 years (*31*).

We used data from the U.S. Behavioral Risk Factor Surveillance System to estimate seasonal influenza vaccination rates among the population of New York City (*33*). In our sensitivity analyses, we evaluated the incremental costs and benefits of raising vaccination rates above this seasonal average.

### Management Algorithms

In our model, the home isolation strategy required persons with FRIs of undetermined origin to remain at home for at least 24 hours after resolution of illness. We assumed that adherence to public health guidelines in the setting of a widespread SARS outbreak would be near universal (*5**, **8*). Under this strategy, we assumed that persons would attempt to manage their illness at home by using self-care, visit a healthcare provider if the illness were serious or persistent, or proceed to a hospital if their illness became progressively severe.

The diagnostic evaluation strategy involved outpatient testing of persons with FRIs to ascertain a microbiologic origin. In this strategy, persons with FRIs of undetermined cause would observe home isolation precautions until the results of diagnostic tests were available. We assumed that a positive SARS RT-PCR test would require isolation precautions for the patient, public health intervention, and additional testing to confirm the diagnosis (*2*). We also assumed that a negative SARS RT-PCR test in conjunction with a positive test for an alternate respiratory pathogen would lead to the elimination of isolation precautions (*2*). If all test results were negative, we assumed that isolation precautions would remain in effect, since current SARS RT-PCR assays are not sufficiently sensitive to rule out SARS (*2*). We also assumed that persons with FRIs, for which the microbiologic origin was confirmed to be due to a pathogen other than SARS-CoV, would return to work only after resolution of their illness.

Under each strategy, we considered the possibility that persons with FRIs seeking medical care might receive antimicrobial drugs during their evaluation. We estimated this probability by using data from the National Ambulatory Medical Care Survey (*32*).

### Illness and Death

Changes in health-related quality of life (HRQL), including the impact of isolation, due to SARS and other FRIs were derived by using the Health Utilities Index Mark 3 (HUI) (*34*). We used the HUI to minimize double counting of productivity losses, since HRQL scores generated from this instrument do not include productivity losses (William Furlong, pers. comm.). Parameters for the HUI were derived from a panel of 4 specialist physicians with clinical experience managing SARS patients in Toronto. These physicians did not directly value health states, but rather functioned as expert "describers," who facilitated the mapping of heath states to community-based preference scores from the HUI.

SARS, influenza, respiratory syncytial virus, and community-acquired pneumonia due to typical and atypical bacteria were assumed to be the primary contributors of death from FRIs on a population level. Mortality data for community-acquired pneumonia were obtained from the National Center for Health Statistics (*35*); data for SARS, influenza, and respiratory syncytial virus were obtained from the medical literature (*24**,**26**,**36*). We estimated that patients with SARS would each transmit infection to 3 other persons if appropriate isolation precautions were not observed (e.g., false-negative SARS RT-PCR test combined with a false-positive test for an alternate diagnosis) (*9**,**10*).

### Costs and Charges

Costs attributable to transportation, ambulatory care (*13*), laboratory tests (*16*), influenza vaccination (*11*), antimicrobial agents (*12*), hospitalization (*14**,**15*), public health investigation (*5**,**18*), and patient time (*17*) were included in the analysis. Transportation costs to see a medical provider were derived by using U.S. national data and were adjusted to account for the estimated proportion of the population driving, using public transportation, or traveling by other means such as biking or walking. The base cost of an ambulatory care visit was estimated by using the national average 2000 Medicare reimbursement rates for a focused medical evaluation (CPT-code 99213); the cost of the rapid influenza test was derived from the Centers for Medicare and Medicaid Services (*16*). The costs of the SARS RT-PCR assay and the multiplex PCR assays used to detect the common respiratory pathogen panel were obtained from a test manufacturer and included 15 minutes of technician time (Prodesse Inc., pers. comm.) (*18*).

Influenza vaccination and antimicrobial drug costs were obtained by using average wholesale prices of pharmaceuticals (*11**,**12*). The costs and frequency of adverse reactions to influenza vaccination were estimated from the medical literature and incorporated into the net costs and benefits of the vaccine (*37*).

Hospital charges and the average length of stay for patients with influenza and other respiratory infections requiring hospitalization were estimated from the Healthcare Cost and Utilization Project (*14*). The Medicare Provider Analysis and Review system was used to derive cost-to-charge ratios and subsequently convert hospital charges into societal costs (*38*). Per diem hospitalization costs for SARS were approximated by using ICD-9 code 769, "respiratory distress syndrome," which was subsequently multiplied by the average length of stay for hospitalized patients with SARS (*15*). Public health costs, including contact investigation, were estimated from the Toronto SARS experience (*5*).

Patient time costs were estimated from data on the median salary of persons living in New York City and included time spent in travel and receiving medical care (*17*). When applicable, medical and nonmedical costs were adjusted to 2004 U.S. dollars by using the Consumer Price Index. The potential economic effects of a SARS outbreak on tourism or other commercial industries were not considered in the analysis.

## Results

If SARS were to resurface during the 2004–2005 respiratory season and the timely establishment of epidemiologic links between SARS cases was not possible, our analysis estimates that the societal costs for New York City would exceed $2.0 billion for each month in which the SARS outbreak and respiratory season coincided.

In our base-case analysis, we found the use of multiplex PCR assays to detect infections with a broad panel of common respiratory pathogens to be the dominant strategy, saving $79 million and resulting in the gain of 8,474 quality-adjusted life-years (QALYs) relative to a strategy of home isolation. If SARS RT-PCR testing were used in conjunction with multiplex PCR assays in our base-case scenario, however, we estimate that costs would increase by about $87 million and have lower effectiveness than multiplex PCR testing alone. These findings are directly related to the very low positive predictive value of the SARS RT-PCR test under low prevalence conditions and the harm resulting from false-positive test results.

If SARS testing were unavailable, confirming an alternate diagnosis for an FRI would be the most effective and least expensive strategy, dominating a strategy of influenza testing alone or home isolation. However, if multiplex PCR testing were also unavailable, home isolation would be the least expensive strategy, albeit less effective than testing for influenza alone. Rapid influenza testing would be accomplished at an incremental cost of $9.0 million but would result in gains of 5,286 QALYs (incremental cost-effectiveness ratio of $1,702 per QALY gained). If the described outbreak were to unfold, a campaign to increase influenza vaccination rates among the general population before the onset of respiratory season would save an estimated $5.0 million and lead to the gain of 128 QALYs for each percentage of New York City's population vaccinated above the seasonal baseline.

**Figure 1:**
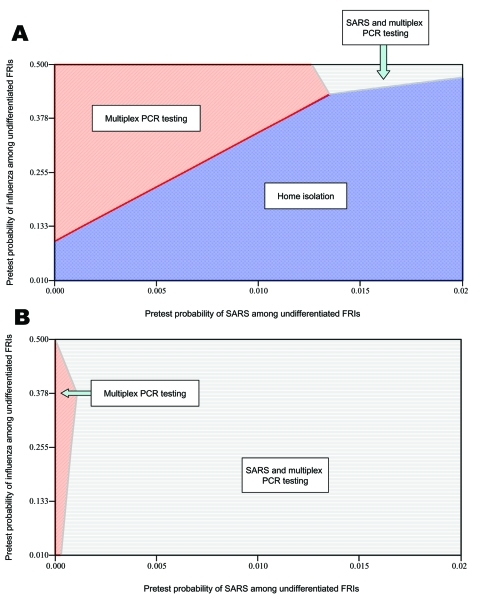
Two-way sensitivity analysis on the prevalence (i.e., pretest probability) of severe acute respiratory syndrome and influenza among undifferentiated febrile respiratory illnesses. A) Preferred strategies to minimize societal costs. B) Preferred strategies to maximize societal health.

**Figure 2:**
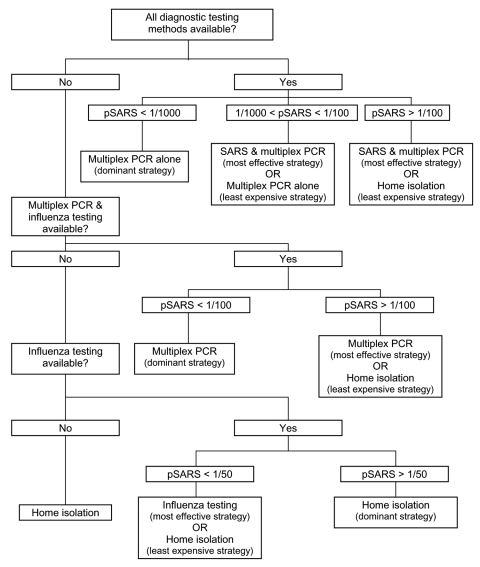
Optimal management of undifferentiated febrile respiratory illnesses under different testing capabilities. pSARS, prevalence (i.e., pretest probability) of severe acute respiratory syndrome among febrile respiratory illnesses. Values are rounded to the nearest fraction.

The total costs, the number of QALYs gained, and the incremental cost-effectiveness of each strategy in the model is shown in Table 3. The results of sensitivity analyses are shown in Table 4 and Figure 1. Algorithms outlining optimal treatment strategies under different testing capabilities are shown in Figure 2.

**Table 3:** Cost-effectiveness of strategies for managing FRIs of undetermined etiology*

Available public health strategies	Monthly total		
	Costs ($ billion)†	QALY gained	Incremental cost-effectiveness (cost per QALY gained)
Home isolation	2.13	0	–
Influenza testing	2.14	5,286	$1,702
Home isolation	2.13	0	–
Influenza testing	2.14	5,286	Dominated
Multiplex RT-PCR testing‡	2.05	8,474	Savings
Home isolation	2.13	0	–
SARS + influenza testing	2.19	5,280	Dominated
Influenza testing	2.14	5,286	Dominated
SARS + multiplex RT-PCR testing‡	2.14	8,429	Dominated
Multiplex RT-PCR testing‡	2.05	8,474	Savings

*FRI, febrile respiratory illness; QALY, quality-adjusted life-year; RT-PCR, reverse transcription–polymerase chain reaction; –, reference category. †Shown in 2004 U.S. dollars rounded to the nearest 10 million. ‡Multiplex RT-PCR testing to detect influenza viruses A and B, respiratory syncytial viruses A and B, parainfluenza viruses 1–3, human metapneumovirus, *Bordetella pertussis*, *Chlamydia pneumoniae*, *Mycoplasma pneumoniae*, *Legionella pneumophila*, and *L. micdadei*.

**Table 4:** Threshold values from one-way sensitivity analyses*

SARS prevalence (%)†	Appropriate strategy
Broad testing capabilities‡	
<0.1%	Multiplex§ RT-PCR testing alone is the most effective and least expensive (i.e., dominant) strategy.
0.1%–0.9%	Combination of SARS and multiplex§ RT-PCR testing is the most effective strategy, while multiplex PCR testing alone is the least expensive strategy.
>0.9%	Combination of SARS and multiplex‡ RT-PCR§ testing is the most effective strategy, while home isolation is the least expensive strategy.
Intermediate testing capabilities¶	
<0.9%	Multiplex§ RT-PCR testing alone is the most effective and least expensive (i.e., dominant) strategy.
>0.9%	Multiplex§ RT-PCR testing alone is the most effective strategy, while home isolation is the least expensive strategy.
Minimal testing capabilities#	
<1.9%	Rapid influenza testing is more effective than home isolation.
Any	Home isolation is less expensive than rapid influenza testing.
Influenza is >36% of FRIs	Rapid influenza testing is the dominant strategy.

*SARS, severe acute respiratory syndrome; FRI, febrile respiration illness; RT-PCR, reverse transcriptase–polymerase chain reaction. †Prevalence or pretest probability of SARS among circulating FRIs. ‡Capable of performing rapid influenza antigen detection tests, multiplex polymerase chain reaction assays to detect influenza viruses A and B, respiratory syncytial viruses A and B, parainfluenza viruses 1–3, human metapneumovirus, *Bordetella pertussis*, *Chlamydia pneumoniae*, *Mycoplasma pneumoniae*, *Legionella pneumophila*, and *L. micdadei*, and coronavirus assays to detect SARS-associated coronavirus and coronaviruses OC43 and 229E (with test turnaround times <24 hours). §Refers to influenza viruses A and B, respiratory syncytial viruses A and B, parainfluenza viruses 1–3, human metapneumovirus, *B. pertussis*, *C. pneumoniae*, *M. pneumoniae*, *L. pneumophila*, and *L. micdadei*. ¶Capable of performing rapid influenza antigen detection tests, multiplex PCR assays to detect influenza viruses A and B, respiratory syncytial viruses A and B, parainfluenza viruses 1–3, human metapneumovirus, *B. pertussis*, *C. pneumoniae*, *M. pneumoniae*, *L. pneumophila*, and *L. micdadei* (with test turnaround times of <24 hours). #Capable of performing rapid influenza antigen detection tests (with test turnaround times of <24 hours).

## Discussion

Our analysis indicates that current diagnostic limitations in discriminating SARS from other common FRIs could have enormous public health and economic consequences, particularly if epidemiologic links between SARS cases were to become tenuous. Under such conditions, we found that most costs would not be related to SARS infections themselves, but rather to procedural changes in the management of other FRIs due to the known or perceived presence of SARS.

We report 3 key findings with direct policy relevance. First, in our base analysis, the most efficient mechanism for discriminating SARS infections from other FRIs involves excluding SARS by confirming an alternate diagnosis. This approach is the most cost-effective strategy under low prevalence conditions since the positive predictive value of SARS RT-PCR tests would be extremely low, and false-positive SARS tests would have deleterious societal repercussions. While the Centers for Disease Control and Prevention supports an approach of excluding SARS by confirming an alternate diagnosis (*2*), caution is advised since SARS coinfection with other respiratory pathogens, including the human metapneumovirus, has been documented (*39*).

Second, we demonstrate that SARS testing under low prevalence conditions would be detrimental from both a public health and an economic perspective. In our analysis, the low positive predictive value of the SARS RT-PCR test translates into unnecessary costs from diagnostic testing, public health interventions, and lost opportunity costs for persons with false-positive test results. Moreover, negative consequences on quality of life would occur when persons are incorrectly diagnosed as having an infection with SARS. Our sensitivity analyses indicate that SARS diagnostic testing should not be performed unless the prevalence or pretest probability of SARS among persons presenting with FRIs exceeds 0.1%.

Third, the use of influenza vaccination as a means to distinguish SARS from influenza has been debated (*40*). In our analysis, we find that if SARS reemerged during respiratory season, higher rates of influenza vaccination among the general population would lead to both health benefits and economic savings. These savings would occur by reductions in influenza illness and death, reductions in costs related to the investigation and isolation of persons with FRIs, and increases in the pretest probability of SARS and, therefore, the positive predictive value of SARS diagnostic testing. The policy implications of these findings, however, must be carefully considered in the context of available influenza vaccine supplies and must ensure their prioritization for groups at high risk (*40*).

Our analysis has several limitations. Foremost was our inability to derive specific estimates of the proportion of FRIs due to specific pathogens. Since the seasonal composition of respiratory viruses and bacteria varies across regions and seasons, we attempted to derive estimates that best reflected seasonal averages. Although national surveillance data on influenza are available, information on other common respiratory pathogens are more limited, since most of these pathogens are self-limited, nonreportable diseases, for which treatment is infrequently sought.

We estimated the sensitivity of current SARS RT-PCR assays to be ≈70% (*4*); however, we recognize that the type of specimen tested and the timing of collection can influence the test's sensitivity (*4**,**36*). In our base-case scenario, in which SARS represented 0.01% of all circulating FRIs, changes in SARS RT-PCR test sensitivity had a negligible impact on overall societal costs and population health. If the pretest probability of SARS were to increase substantially above our baseline, however, SARS RT-PCR test sensitivity would have an increasingly important influence on the effectiveness of strategies involving SARS testing.

Our reported test sensitivity for the multiplex PCR assays, which detect common respiratory viruses and bacteria, is lower than values reported in the medical literature (*20**–**22*). Since estimates in the literature reflect experimental conditions and are essentially measures of test efficacy, we wished to estimate real-world effectiveness of these tests by taking into account factors such as ineffective specimen collection methods, delays in laboratory testing, or other related factors.

Our analysis demonstrates that influenza vaccination would lead to cost-savings, which has been reported in other studies of healthy adults in the pre-SARS era (*31**,**37*). However, the specific benefits quantified in our analysis would only be realized if the conditions of the model were to occur, i.e., the reemergence of SARS during a respiratory season, when epidemiologic links between cases are poorly defined.

Finally, our analysis does not adequately address the complexities of microbiologic coinfection in the development of FRIs. While our model allows for multiple positive test results, we assume that only 1 organism is responsible for causing an FRI. This issue is particularly relevant when considering SARS coinfection with other respiratory organisms (*39*). Nonetheless, in our analysis the effect of SARS coinfection on a population level is minimal given that SARS-CoV infections make up only 0.01% of all FRIs.

Speculation about the reemergence of SARS has prompted heightened surveillance by health officials worldwide. Given that SARS has resurfaced in each of the past 2 respiratory seasons in the absence of accidental laboratory exposures, SARS-CoV may reappear annually at times when FRIs are widely prevalent among the general population. Even if the world does not experience another large-scale, multinational outbreak, healthcare providers around the globe will continue to see patients with nonspecific FRIs who are incidentally returning from SARS-affected areas. This fact underscores the importance of having evidence-based guidelines to facilitate the timely and accurate distinction of SARS infections from other FRIs of lesser public health importance. Our analysis provides guidance on the most effective and efficient use of resources when managing persons with FRIs of undetermined etiology when the epidemiologic history for SARS is either unavailable or unreliable. Our findings will help policy makers and healthcare practitioners make decisions based on available evidence and avoid decisions that are driven by fear and misinformation.
